# Crossmodal Recruitment of the Ventral Visual Stream in Congenital Blindness

**DOI:** 10.1155/2012/304045

**Published:** 2012-06-14

**Authors:** Maurice Ptito, Isabelle Matteau, Arthur Zhi Wang, Olaf B. Paulson, Hartwig R. Siebner, Ron Kupers

**Affiliations:** ^1^École d'Optométrie, Université de Montréal, Montréal, QC, Canada H3T 1P1; ^2^Department of Neuroscience and Pharmacology, Panum Institute, University of Copenhagen, Blegdamsvej 3B, 2200 Copenhagen, Denmark; ^3^Danish Research Centre for Magnetic Resonance, Copenhagen University Hospital Hvidovre, 2650 Hvidovre, Denmark; ^4^Department of Radiology, Beijing Hospital, 100054 Beijing, China; ^5^Neurobiology Research Unit, Rigshospitalet, Copenhagen University Hospital, 2100 Copenhagen, Denmark

## Abstract

We used functional MRI (fMRI) to test the hypothesis that blind subjects recruit the ventral visual stream during nonhaptic tactile-form recognition. Congenitally blind and blindfolded sighted control subjects were scanned after they had been trained during four consecutive days to perform a tactile-form recognition task with the tongue display unit (TDU). Both groups learned the task at the same rate. In line with our hypothesis, the fMRI data showed that during nonhaptic shape recognition, blind subjects activated large portions of the ventral visual stream, including the cuneus, precuneus, inferotemporal (IT), cortex, lateral occipital tactile vision area (LOtv), and fusiform gyrus. Control subjects activated area LOtv and precuneus but not cuneus, IT and fusiform gyrus. These results indicate that congenitally blind subjects recruit key regions in the ventral visual pathway during nonhaptic tactile shape discrimination. The activation of LOtv by nonhaptic tactile shape processing in blind and sighted subjects adds further support to the notion that this area subserves an abstract or supramodal representation of shape. Together with our previous findings, our data suggest that the segregation of the efferent projections of the primary visual cortex into a dorsal and ventral visual stream is preserved in individuals blind from birth.

## 1. Introduction

It is well established that early-onset blindness leads to widespread neuroplastic changes. For instance, studies have shown that the senses of hearing and touch are more developed in blind than sighted individuals [1–6], probably due to training-induced plasticity. The enlargement of the somatic and motor area representation of the index finger in proficient Braille readers is a clear example of this experience-dependent plastic process [7]. Brain imaging studies using 18F-fluoro-deoxyglucose-positron emission tomography (FDG-PET) have shown that, despite the absence of visual input, the occipital cortex of congenitally blind individuals shows a supranormal metabolism at rest [8, 9]. This indicates that the visually deprived cortex is still functionally active and can be recruited by other modalities such as touch, hearing, and smell. Indeed, studies using a variety of brain imaging tools such as PET, functional, magnetic resonance imaging (fMRI), event-related potentials and magnetoencephalography, all concur on a recruitment of the visual cortex of early blind individuals during various nonvisual tasks (e.g., [10–14]). 

Numerous brain imaging studies have consistently found activations of occipital cortical areas when blind subjects perform a range of tactile tasks such as Braille character recognition, vibrotactile discrimination, and haptic object exploration [15–20]. Previous work from our laboratory showed that blind subjects who had been trained to use the TDU in an orientation and motion discrimination task [13, 14] or in a navigation task [21] activate their visual cortex. The observed activation patterns within the visual cortex were remarkably similar to those observed in sighted individuals performing corresponding visual tasks. The modularity of these activations is further substantiated by the observation that in early blind individuals, transcranial magnetic stimulation (TMS) of the reorganized visual cortex elicits somatotopically organized tactile sensations [22, 23]. TMS studies have also provided evidence for the functional implication of the occipital cortex in tactile processing. For example, the demonstration that repetitive TMS of the occipital cortex disrupts Braille reading performance in the blind [24, 25] suggests that the contribution of the visually-deprived occipital cortex to nonvisual functions is indeed functionally relevant. Together, these findings indicate that the visually deprived posterior cortical regions are much more adaptable than previously thought and may act either as a high-level multisensory area [26] or undergoes a cross-modal plastic reorganization [27]. 

The visual system is grossly subdivided into a dorsal and a ventral processing stream [28]. Area hMT+, a critical part of the dorsal visual pathway, involved in visual motion processing, is recruited during tactile and auditory motion discrimination task in early blind subjects [14, 29–31], suggesting that the dorsal “where” processing stream is functionally preserved in subjects lacking vision from birth. This raises the question whether the ventral processing stream, known to participate in object and shape recognition [30–39], is also preserved in blind subjects. Evidence in support of this hypothesis comes from a study by Pietrini et al. [12] that showed category-specific recruitment of the ventral temporal cortex by haptic exploration of objects in congenitally blind and sighted individuals. Therefore, the aim of this study was to investigate whether the ventral stream will also be activated by nonhaptic exploration of shapes. 

## 2. Materials and Methods

### 2.1. Subjects

Ten sighted control (five females; mean age: 21 ± 11 y.) and eight blind (seven congenitally and one early blind) individuals with no recollection of any visual experience (four females; mean age: 31 ± 10 y.) participated in this study. Causes of blindness were retinopathy of prematurity [7] and Leber's congenital amaurosis [1]. Visual inspection of the structural brain MRI scans by a trained neuroradiologist did not reveal macroscopic abnormalities and none of the subjects had a history of psychiatric or neurological illness. The study protocol was approved by the local ethics committee (Project ID: KF-01328723) and all subjects provided written informed consent.

### 2.2. Electro-Tactile Stimulation of the Tongue

**Figure 1:**
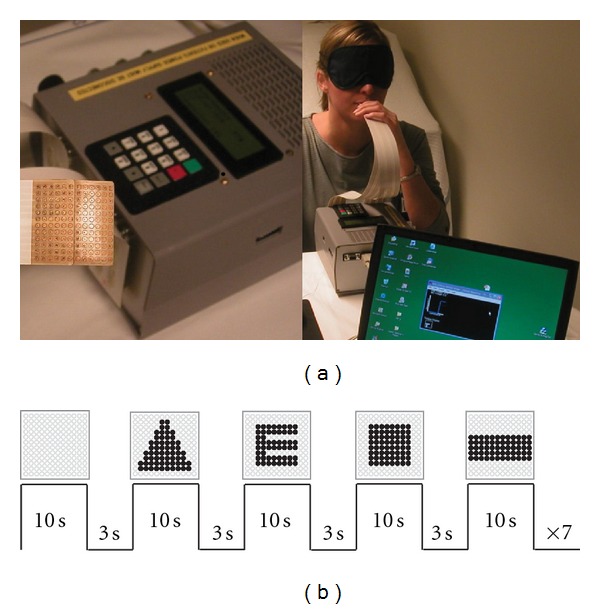
Experimental setup. (a) The tongue display unit (TDU) and its components. (b) The fMRI block design. During each of the two fMRI runs, 7 stimulation blocks were presented. One block consisted of a rest period and Shape stimuli used during training and fMRI.

The apparatus has been described in detail elsewhere [13]. Briefly, it consists of a tongue display unit (TDU, Wicab Inc.), an electrode array (3 × 3 cm) with 144 gold-plated contacts arranged in a 12 × 12 matrix and a laptop with custom-made software (Figure 1(a)). Computer-generated geometric shapes were converted into electrical pulses and delivered to the tongue via the electrode array. Stimulation intensity was controlled by the subject and could be adjusted at any time to allow optimal perception of the stimuli.

### 2.3. Behavioural Training

Both blind and blindfolded control subjects were trained during 10 sessions, stretched over 4 consecutive days. Each session lasted around 15 minutes and comprised 28 trials. During training, subjects learned to use the TDU to recognize four different shapes that were randomly presented: a square, a triangle, a rectangle, and the letter E (Figure 1(b)). Participants were given a maximum of 30 seconds to identify each stimulus and they received immediate feedback about the correctness of their response. Both the reaction time and the response accuracy were measured. It was stressed that correctness of responses was more important than speed. Stimuli were presented statically and participants could not explore the images by using exploratory movements of a computer mouse as was the case in our previous PET study on orientation discrimination [13]. Prior to the training sessions, participants were familiarized with the TDU and the experimental procedures. They were told the forms that were going to be used and that their task was to correctly identify the shape that was presented. Blind participants were asked whether they were familiar with the shapes that were going to be presented and they were given the opportunity to explore haptically plastic copies of the four shapes if necessary. Training sessions were limited to a maximum of 15 minutes to avoid habituation to tongue stimulation. Participants were given two or maximum 3 training sessions per day. Between two successive sessions there was a minimum time interval of 30 minutes. We chose to work with a limited set of shapes in order not to overload memory and cognitive processing since during the fMRI session. Subjects had to indicate their response by pressing one of the four keys on a response pad (1: square; 2: triangle; 3: rectangle and; 4: letter E). All participants were trained by the same experimenter (IM). The criterion for successful learning was set to 85% correct responses in two consecutive sessions. Participants who reached this criterion could participate the next day in the fMRI examination. Statistical analysis of the behavioural data was carried out using ANOVA (SPSS18, Chicago, Ill, US). Values of *P* < 0.05 were considered as statistically significant.

### 2.4. MRI Experimental Design

Following behavioural training, subjects performed the shape recognition task during whole-brain fMRI. We used an fMRI block design with periods of rest (the electrode array was placed on the tongue but no electrotactile stimulation was administered) and task (i.e., nonhaptic shape recognition). The same shapes were presented in the fMRI session as during behavioural training. Two fMRI runs were carried out, each lasting 7 minutes and 40 seconds. Each run consisted of alternating rest and task blocks (Figure 1(b)). During a task block, four stimuli, one for each form, were presented in a random order. This was repeated seven times, resulting in 35 blocks per fMRI run. Each stimulus lasted 10 s and was followed by a 3 s interval during which subjects had to indicate which form had been presented by pressing one of 4 buttons on a keypad with their right hand. Each button corresponded to one of the stimulus forms. Prior to scanning, subjects practiced to use the appropriate corresponding response button. 

### 2.5. Image Acquisition and Analysis

Task-related changes in the (blood oxygenation level-dependent BOLD signal were measured with whole-brain fMRI using a Siemens Trio 3 Tesla MR Scanner (Siemens, Erlangen, Germany), equipped with an 8-channel head coil. The multislice gradient echo-planar imaging sequence had a repetition time (TR) = 2500 ms, echo time (TE) = 50 ms, flip angle (FA) = 90°, and field of view (FOV) of 192 mm (matrix: 64 × 64). Each volume consisted of 42 slices in an inclined axial plane, aligned to the AC-PC axis, with a slice thickness of 4 mm, resulting in a voxel size of 4 × 4 × 4 mm. A total of 368 functional brain volumes were acquired per subject. After the fMRI session, a high-resolution structural T1-weighted three-dimensional brain scan (MPRAGE) was acquired using a gradient echo pulse sequence (TE = 9.20 ms; flipangle = 30°; FOV = 256 mm; matrix = 256 × 256; voxelsize = 1 mm^3^). 

The MRI data were analyzed using Statistical Parametric Mapping software (SPM5, Wellcome Department of Cognitive Neurology, London, UK). Functional volumes were motion-corrected using SINC interpolation and spatially normalized to the reference space defined by the MRI template supplied by the Montreal Neurological Institute (MNI). Images were spatially smoothed with an 8-mm wide Gaussian kernel to improve the signal-to-noise ratio. 

For the statistical analysis, active conditions were fitted with a box-car function convolved with the hemodynamic response function. Low-frequency temporal drifts were removed by applying a 128-s high-pass filter. The duration of all conditions was modelled, except for the 10 s rest periods, which served as baseline. In order to estimate the effects associated with the experimental design, we evaluated BOLD signal changes associated with the contrast active task (shapes) compared to the control task (rest). Following single subject analyses, we performed a random-effect analysis within and between groups using the individual contrast estimates for each functional run. Activation maps were thresholded at *P* < 0.01, corrected for multiple comparisons using the false discovery rate (FDR) [40]. We applied a conservative extent threshold of 20 contiguous voxels. 

## 3. Results

### 3.1. Behavioural Training

**Figure 2:**
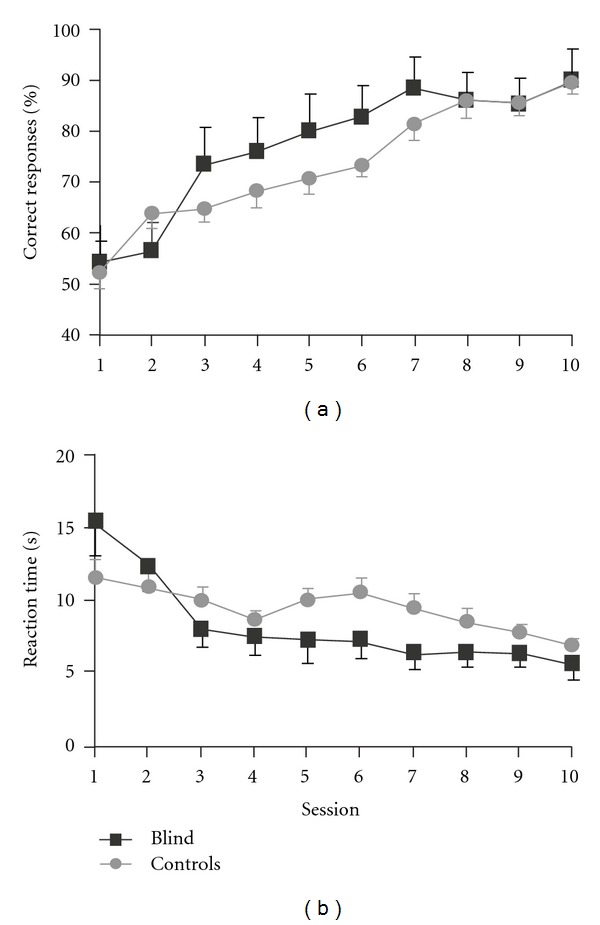
Learning curves for shape recognition in congenitally blind and blindfolded control subjects. (a) Mean percentage changes ± SEM of correct responses and (b) mean reaction times ± SEM. No significant differences in performance were observed between the groups.

Both blind and sighted control subjects learned the tactile form discrimination task within the 10 sessions.  Figure 2 illustrates the learning curves for percentage of correct responses and reaction times. A statistical analysis of the time × group interactions yielded no significant differences in the percentage of correct responses (*F* = 0.728; *P* > 0.05) or reaction times at the end of the training (*F* = 1.016; *P* > 0.05). 

### 3.2. Functional MRI

**Figure 3:**
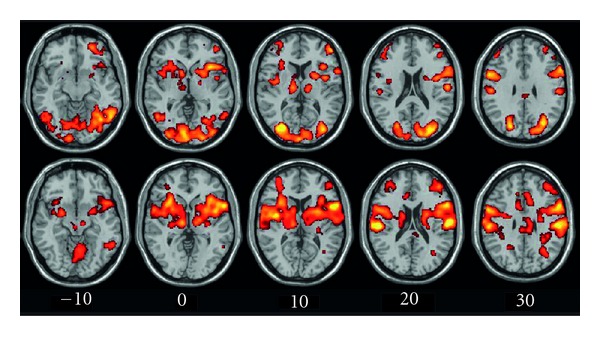
Axial maps showing brain activations for the contrast “shapes-rest” in congenitally blind (upper row) and blindfolded control (lower row) subjects. The color-coded t-maps illustrate voxels showing a task-related increase in activation at *P* < 0.01, FDR-corrected. Right side of the brain is to the right of the image.

**Figure 4:**
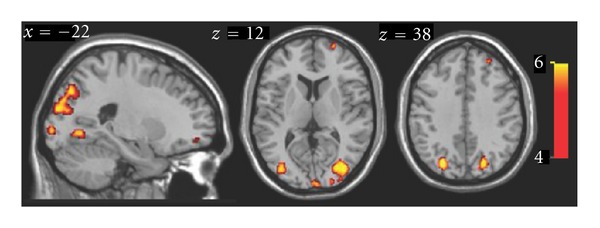
Cortical maps showing brain areas where activity was significantly larger in congenitally blind compared to blindfolded control subjects. The color-coded t-map shows the voxels with a relative increase in task-related activation in the blind group relative to controls at *P* < 0.01, FDR-corrected. Right side of the brain is to the right of the image.

Blind subjects but not blindfolded sighted controls activated large areas of occipital (cuneus, inferior and middle occipital gyri and lingual gyrus) and occipito-temporal (fusiform gyrus) cortices (Figure 3). Both blind and sighted controls showed increased BOLD responses in the inferotemporal cortex (including area LOtv), post-central gyrus, superior and inferior parietal lobule, precuneus, prefrontal cortex, cingulate gyrus, insula, and cerebellum. Task-related activations for blind and control subjects are listed in Table 1. A direct comparison of the activation maps in both groups showed that BOLD increases in the inferior temporal gyrus, middle occipital cortex, and precuneus were significantly stronger in blind subjects (Figure 4). In contrast, blindfolded-sighted control subjects showed a relative larger BOLD response increase in the right postcentral gyrus (BA3) and the left anterior cingulate cortex (BA24) only (data not shown). We also observed activation in both blind and controls in left and right premotor areas that are probably due to the subject's preparation to respond to the tactile stimulation. An increased BOLD response was also found in bilateral somatosensory cortex for both groups of subjects. 

## 4. Discussion

In this study, we report that congenitally blind but not sighted subjects activated large parts of the occipital cortex when performing a nonhaptic shape recognition task. Our data further showed that both groups recruited the inferotemporal cortex, including area LOtv, in response to 2D tactile shape information extracted from electrotactile stimulation of the tongue. Previous studies showed that area LOtv processes form information in the absence of visual input through haptic [12] or auditory modalities [15, 30]. The present data extend these findings by showing that area LOtv processes form information even when tactile stimuli are delivered nonhaptically and to a body part, like the tongue, that is not primarily devoted to shape recognition.

### 4.1. Activation of IT/LOtv Complex

We found strong task-related activation along the occipital/inferior temporal cortical border in both sighted and blind subjects. Whereas IT/LOtv was activated bilaterally in blind subjects, it was activated only in the right hemisphere in sighted participants. This might be due to the relatively small sample size. Indeed, when using a less stringent criterion for statistical significance (*P* < 0.01, uncorrected), an increased BOLD response was also noted in the left hemisphere. Moreover, a conjunction analysis of the activation patterns in both groups confirms the bilateral activation of IT/LOtv although the cluster size was markedly larger in the right compared to the left hemisphere (data not shown)The activation pattern in both groups encompassed a region that Amedi and coworkers [36, 37] have coined the lateral occipital tactile visual area. The stereotactic coordinates of our LOtv activation in both groups (see Table 1) are very close to those reported by others [12, 36, 41]. LOtv is a subregion within the human lateral occipital cortex (LOC) that is robustly activated during both visual and tactile object recognition. Amedi and coworkers [37] demonstrated that for both modalities, LOtv has a preference for objects compared to textures and scrambled objects; this area is only weakly activated by the motor, naming and visual imagery components of object recognition [37]. Area LOtv is also recruited by tactile exploration of novel, meaningless three-dimensional clay objects, suggesting that it responds more to form than to semantic features of objects [39]. Our finding of LOtv activation in both groups during the presentation of tactile stimuli is in accordance with previous results reported in normally sighted [12, 36, 36, 42–44] and blind [12] participants. The results further show for the first time that not only three-dimensional tactile stimuli but also two-dimensional nonhaptic tactile information can recruit area LOtv, adding further support that this area subserves an abstract or supramodal representation of shape information [45].

### 4.2. Occipital Cortex

Only blind subjects showed a significant BOLD response in several regions within the occipital cortex including the cuneus, the lingual gyrus and the inferior, middle, and superior occipital gyri. The activation pattern in the blind following training shows remarkable similarities with that observed in normal seeing subjects during the performance of visual form discrimination tasks (see review by [45]). Pietrini and coworkers [12] reported activations in portions of the ventral stream such as the lingual and fusiform gyri and inferior occipital cortex during haptic object recognition in blind subjects. Other studies have shown that the visual cortex in blind subjects can also be recruited by auditory and olfactory stimuli and cognitive processes [38, 46–48] providing further evidence that the visual cortex can be reorganized to mediate a variety of non-visual functions in the blind.

Another issue is the potential role of mental imagery in the visual cortex activation. A number of previous brain imaging studies on haptic processing [49, 50] and auditory-based sensory substitution [51] in blindfolded sighted subjects have suggested that neural activity related to visual imagery may account for the activation in the occipital cortex. For the following reasons, it is unlikely that visual imagery explains the current findings. First, our blind subjects never had visual experiences and during the debriefing following the experiments, they did not report that they had engaged in visual imagery during the orientation detection task (see also [12]). Secondly, if mental imagery would be at the basis of the activation in the occipital cortex, sighted controls should activate the visual cortex to a larger extent compared to congenitally blind participants, which was clearly not the case. Finally, the question whether congenitally blind subjects have true “visual imagery” (instead of imagery) remains a matter of debate [52].

In accordance with several other studies (e.g., [13, 14, 53, 54]), we report here a lack of activation in the occipital cortex of our blindfolded controls. Previous neuroimaging studies in normally sighted subjects have yielded inconsistent results regarding the implication of V1 in tactile processing: some studies showing no activation of V1, others showing activation of V1 only [18, 55], activation of V1 accompanied by a deactivation of extrastriate areas [56], or activation of extrastriate cortical areas only [12]. Of note, most studies showing V1 activation in tactile processing in normal subjects used 3D stimuli that were palpated haptically with the hand or fingers. In our study, we used 2-D shape stimuli presented passively to the tongue and thus, required no active haptic exploration. 

### 4.3. Possible Mechanisms for Cross-Modal Responses

A critical question in the study of cross-modal processing in the blind is whether the recruitment of the occipital cortex occurs through changes of existing neural network or through the formation of new neural connections. In this study, as well as in our previous studies using the same sensory substitution device, cross-modal responses were already observed after only a four to seven day period of intensive training [13, 22]. The speed with which these neuroplastic changes occur suggest that they are mediated by the unmasking or strengthening of preexisting cortico-cortical connections [13, 22]. The observed striate and extrastriate activations in the blind have been attributed to a cortico-cortical feedback pathway from primary somatosensory cortex (S1) through the posterior parietal cortex [22, 23]. The posterior parietal cortex is a highly multimodal association area. Investigations in macaque and humans have demonstrated that the anterior intraparietal area (AIP) and ventral intraparietal area (VIP) are likely regions where visuotactile multimodal information of object features and motion processing is integrated in sighted participants. Neurons in the macaque AIP, for instance, are sensitive to three-dimensional features of objects such as size, shape, and orientation during object manipulation under visual control [57, 58]. Neuroimaging studies in humans have also demonstrated recruitment of AIP during tactile shape processing [59, 60] and during orientation discrimination of visual stimuli [13, 61]. In blind subjects, who lack bottom-up visual processing, tactile inputs from these multimodal areas may then lead to a recruitment of the visual cortex via these multimodal areas. This assumption is supported by the strong activation of the posterior parietal cortex observed in the blind in the present study and is moreover reinforced by the results of several additional neuroimaging studies [13, 17, 38]. This hypothesis is also in line with a recent report that used dynamic causal modeling of fMRI data to investigate the cross-modal plasticity of effective connectivity in the blind during a Braille reading task [62]. It is also possible that new aberrant subcortical projections could be responsible for the evoked activity in the visual cortex of congenital blind individuals. For example, animal models of bilateral enucleation in hamsters [60], congenital blindness in mice [63, 64], and natural very low vision like the blind mole rat [65] have indicated the formation of new ectopic projections from the inferior colliculus to the lateral geniculate nucleus, the thalamic primary visual relay. More advanced methods, such as functional connectivity analysis, will be helpful to better understand through which pathways nonhaptic tactile information is funnelled to the visual cortex of the blind.

### 4.4. Methodological Considerations

The main limitation of this study is the sample size. While eight subjects are considered to be a relatively small sample size for a classical fMRI study, we would like to emphasize that congenitally blind individuals represent an exceptionally rare population, even more so when strict selection requirements are enforced, as in this study. We would further like to stress that sample sizes of congenitally blind individuals reported in most fMRI studies in the literature are similar or smaller as compared to the present one [12–18, 29–31, 38, 39, 54, 55, 62, 66]. Larger numbers of subjects certainly are required to make rigorous statistical comparisons between the sighted and congenitally blind groups in terms of distribution and extent of brain response to shape recognition following stimulation by TDU. Nevertheless, the data were obtained using a random-effects analysis and FDR-corrected statistical thresholds. A final limitation is that we did not use functional localizer scans in our sighted subjects to identify subregions within the ventral stream. 

## 5. Conclusion

The question we have addressed in this and our previous studies is whether the functional segregation of the visual cortex in a dorsal and ventral visual pathway is preserved in individuals who were born without vision or who lost their sight at a very early age. The present results significantly extend to our previously published data on motion processing via the tongue in the blind [14], showing that both pathways are preserved in this population and add to growing evidence that the visual cortex can be reorganized to mediate non-visual functions in the blind.

## Figures and Tables

**Table 1:** Activation clusters for “shapes versus rest” in blind and sighted subjects.

Anatomical area of activation	BA	Congenitally blind			Sighted controls				
		Talairach coordinates			Talairach coordinates				
		*x*	*y*	*z*	*t*	*x*	*y*	*z*	*t*
*Occipital cortex *									
Cuneus	18	−26	−75	15	8.12				
		12	−85	19	6.13				
Lingual gyrus	17	−8	−87	4	5.84				
		8	−85	3	4.38				
Inferior occipital gyrus	19	−48	−80	−3	4.02				
		34	−76	−8	4.78				
Middle occipital gyrus	19	−36	−85	6	4.71				
	37	53	−61	−9	6.36				
Superior occipital gyrus	19	36	−73	24	4.54				
*Temporal cortex *									
Fusiform gyrus	19	−36	−74	−10	3.48				
		38	−64	−5	5.76				
Inferior temporal gyrus (LOtv)	19	−51	−66	−2	4.60				
	37/20	55	−53	−4	5.36	51	−52	−7	4.67
Middle temporal gyrus	37	−50	−60	0	5.41	−55	−51	−4	5.43
		53	−62	0	4.59				
Superior temporal gyrus	22					−57	4	2	6.29
						55	10	3	8.49
*Parietal Cortex *									
Precuneus	31/7	−26	−75	15	8.12	−10	−64	44	5.63
	19/7	26	−70	35	7.57	12	−62	49	7.11
Inferior parietal lobule	40	−46	−39	44	6.96	−44	−31	49	9.73
		42	−31	44	5.92	46	−33	44	8.72
Superior parietal lobule	7	−30	−54	51	6.82	−20	−53	58	5.75
		30	−54	52	6.50	32	−52	49	6.82
Postcentral gyrus	2	−53	−25	40	5.65	−50	−25	44	9.49
	3/2	59	−20	34	5.73	51	−28	53	9.61
*Prefrontal* and *frontal cortices *									
Precentral gyrus	6/44	−61	0	35	4.48	−50	2	11	6.81
		62	5	29	3.73	50	8	9	8.09
Inferior frontal gyrus	9	−55	5	29	5.91	−57	5	26	6.98
	47/9	34	17	−3	6.02	53	9	28	7.58
Middle frontal gyrus	6	−26	−2	46	4.87	−38	−3	54	5.87
		30	−1	48	6.37	40	2	50	7.00
Medial frontal gyrus	6					−2	−1	50	6.63
						4	1	50	6.52
Superior frontal gyrus	6					−18	5	62	4.33
						18	5	62	5.08
*Cingulate* and *Insular cortices *									
Cingulate gyrus	32	2	10	42	6.31	2	10	42	8.53
Insula	13	−40	5	13	4.76	−34	14	1	8.28
		38	3	15	5.43	34	21	3	7.55
*Cerebellum *		−32	−74	−22	5.36	−28	−67	−15	8.16
		4	−73	−13	4.34	28	−67	−15	6.35

## References

[1] Collignon O, Charbonneau G, Lassonde M, Lepore F (2009). Early visual deprivation alters multisensory processing in peripersonal space. *Neuropsychologia*.

[2] Chebat DR, Chen JK, Schneider F, Ptito A, Kupers R, Ptito M (2007). Alterations in right posterior hippocampus in early blind individuals. *NeuroReport*.

[3] Fieger A, Röder B, Teder-Sälejärvi W, Hillyard SA, Neville HJ (2006). Auditory spatial tuning in late-onset blindness in humans. *Journal of Cognitive Neuroscience*.

[4] Goldreich D, Kanics IM (2003). Tactile acuity is enhanced in blindness. *Journal of Neuroscience*.

[5] Goldreich D, Kanics IM (2006). Performance of blind and sighted humans on a tactile grating detection task. *Perception and Psychophysics*.

[6] Van Boven RW, Ingeholm JE, Beauchamp MS, Bikle PC, Ungerleider LG (2005). Tactile form and location processing in the human brain. *Proceedings of the National Academy of Sciences of the United States of America*.

[7] Pascual-Leone A, Torres F (1993). Plasticity of the sensorimotor cortex representation of the reading finger in Braille readers. *Brain*.

[8] De Volder AG, Bol A, Blin J (1997). Brain energy metabolism in early blind subjects: neural activity in the visual cortex. *Brain Research*.

[9] Kupers R, Pietrini P, Ricciardi E, Ptito M (2011). The nature of consciousness in the visually-deprived brain. *Frontiers in Psychology*.

[10] Merabet LB, Rizzo JF, Amedi A, Somers DC, Pascual-Leone A (2005). What blindness can tell us about seeing again: merging neuroplasticity and neuroprostheses. *Nature Reviews Neuroscience*.

[11] Noppeney U (2007). The effects of visual deprivation on functional and structural organization of the human brain. *Neuroscience and Biobehavioral Reviews*.

[12] Pietrini P, Furey ML, Ricciardi E (2004). Beyond sensory images: object-based representation in the human ventral pathway. *Proceedings of the National Academy of Sciences of the United States of America*.

[13] Ptito M, Moesgaard SM, Gjedde A, Kupers R (2005). Cross-modal plasticity revealed by electrotactile stimulation of the tongue in the congenitally blind. *Brain*.

[14] Ptito M, Matteau I, Gjedde A, Kupers R (2009). Recruitment of the middle temporal area by tactile motion in congenital blindness. *NeuroReport*.

[15] Amedi A, Stern WM, Camprodon JA (2007). Shape conveyed by visual-to-auditory sensory substitution activates the lateral occipital complex. *Nature Neuroscience*.

[16] Büchel C, Price C, Frackowiak RSJ, Friston K (1998). Different activation patterns in the visual cortex of late and congenitally blind subjects. *Brain*.

[17] Burton H (2003). Visual cortex activity in early and late blind people. *Journal of Neuroscience*.

[18] Burton H, McLaren DG, Sinclair RJ (2006). Reading embossed capital letters: an fMRI study in blind and sighted individuals. *Human Brain Mapping*.

[19] Sadato N, Pascual-Leone A, Grafman J (1996). Activation of the primary visual cortex by Braille reading in blind subjects. *Nature*.

[20] Sadato N, Pascual-Leone A, Grafman J, Deiber MP, Ibañez V, Hallett M (1998). Neural networks for Braille reading by the blind. *Brain*.

[21] Kupers R, Chebat DR, Madsen KH, Paulson OB, Ptito M (2010). Neural correlates of virtual route recognition in congenital blindness. *Proceedings of the National Academy of Sciences of the United States of America*.

[22] Kupers R, Fumal A, De Noordhout AM, Gjedde A, Schoenen J, Ptito M (2006). Transcranial magnetic stimulation of the visual cortex induces somatotopically organized qualia in blind subjects. *Proceedings of the National Academy of Sciences of the United States of America*.

[23] Ptito M, Schneider FCG, Paulson OB, Kupers R (2008). Alterations of the visual pathways in congenital blindness. *Experimental Brain Research*.

[24] Cohen LG, Celnik P, Pascual-Leone A (1997). Functional relevance of cross-modal plasticity in blind humans. *Nature*.

[25] Kupers R, Pappens M, De Noordhout AM, Schoenen J, Ptito M, Fumal A (2007). rTMS of the occipital cortex abolishes Braille reading and repetition priming in blind subjects. *Neurology*.

[26] Büchel C (2003). Cortical hierarchy turned on its head. *Nature Neuroscience*.

[27] Pietrini P, Ptito M, Kupers R, Laureys S, Tononi  G (2009). Blindness and consciousness: new lights from the dark. *The Neurology of Consciousness*.

[28] Goodale MA, David Milner A (1992). Separate visual pathways for perception and action. *Trends in Neurosciences*.

[29] Poirier C, Collignon O, Scheiber C (2006). Auditory motion perception activates visual motion areas in early blind subjects. *NeuroImage*.

[30] Poirier CC, De Volder AG, Tranduy D, Scheiber C (2006). Neural changes in the ventral and dorsal visual streams during pattern recognition learning. *Neurobiology of Learning and Memory*.

[31] Ricciardi E, Vanello N, Sani L (2007). The effect of visual experience on the development of functional architecture in hMT+. *Cerebral Cortex*.

[32] Haxby JV, Gobbini MI, Furey ML, Ishai A, Schouten JL, Pietrini P (2001). Distributed and overlapping representations of faces and objects in ventral temporal cortex. *Science*.

[33] Matteau I, Kupers R, Ricciardi E, Pietrini P, Ptito M (2010). Beyond visual, aural and haptic movement perception: hMT+ is activated by electrotactile motion stimulation of the tongue in sighted and in congenitally blind individuals. *Brain Research Bulletin*.

[34] Underleider LG, Mishkin M, Ingle MA, Goodale MI, Masfield RJW (1982). Two cortical visual systems. *Analysis of Visual Behavior*.

[35] Farivar R (2009). Dorsal-ventral integration in object recognition. *Brain Research Reviews*.

[36] Amedi A, Jacobson G, Hendler T, Malach R, Zohary E (2002). Convergence of visual and tactile shape processing in the human lateral occipital complex zohary. *Cerebral Cortex*.

[37] Amedi A, Malach R, Hendler T, Peled S, Zohary E (2001). Visuo-haptic object-related activation in the ventral visual pathway. *Nature Neuroscience*.

[38] Amedi A, Raz N, Pianka P, Malach R, Zohary E (2003). Early ’visual’ cortex activation correlates with superior verbal memory performance in the blind. *Nature Neuroscience*.

[39] James TW, Humphrey GK, Gati JS, Servos P, Menon RS, Goodale MA (2002). Haptic study of three-dimensional objects activates extrastriate visual areas. *Neuropsychologia*.

[40] Genovese CR, Lazar NA, Nichols T (2002). Thresholding of statistical maps in functional neuroimaging using the false discovery rate. *NeuroImage*.

[41] Kassuba T, Klinge C, Hölig C (2011). The left fusiform gyrus hosts trisensory representations of manipulable objects. *NeuroImage*.

[42] Prather SC, Sathian K (2002). Mental rotation of tactile stimuli. *Cognitive Brain Research*.

[43] Reed CL, Shoham S, Halgren E (2004). Neural substrates of tactile object recognition: an fMRI study. *Human Brain Mapping*.

[44] Stoesz MR, Zhang M, Weisser VD, Prather SC, Mao H, Sathian K (2003). Neural networks active during tactile form perception: common and differential activity during macrospatial and microspatial tasks. *International Journal of Psychophysiology*.

[45] Amedi A, Von Kriegstein K, Van Atteveldt NM, Beauchamp MS, Naumer MJ (2005). Functional imaging of human crossmodal identification and object recognition. *Experimental Brain Research*.

[46] Röder B, Stock O, Bien S, Neville H, Rösler F (2002). Speech processing activates visual cortex in congenitally blind humans. *European Journal of Neuroscience*.

[47] Gougoux F, Lepore F, Lassonde M, Voss P, Zatorre RJ, Belin P (2004). Neuropsychology: pitch discrimination in the early blind. *Nature*.

[48] Beaulieu-Lefebvre M, Schneider FC, Kupers R, Ptito M (2011). Odor perception and odor awareness in congenital blindness. *Brain Research Bulletin*.

[49] Zhang M, Weisser VD, Stilla R, Prather SC, Sathian K (2004). Multisensory cortical processing of object shape and its relation to mental imagery. *Cognitive, Affective and Behavioral Neuroscience*.

[50] Lacey S, Flueckiger P, Stilla R, Lava M, Sathian K (2010). Object familiarity modulates the relationship between visual object imagery and haptic shape perception. *NeuroImage*.

[51] Poirier C, De Volder A, Tranduy D, Scheiber C (2007). Pattern recognition using a device substituting audition for vision in blindfolded sighted subjects. *Neuropsychologia*.

[52] Kaski D (2002). Revision: is visual perception a requisite for visual imagery?. *Perception*.

[53] Sadato N, Okada T, Honda M, Yonekura Y (2002). Critical period for cross-modal plasticity in blind humans: a functional MRI study. *NeuroImage*.

[54] Harada T, Saito DN, Kashikura KI (2004). Asymmetrical neural substrates of tactile discrimination humans: a functional magnetic resonance imaging study. *Journal of Neuroscience*.

[55] Saito DN, Okada T, Honda M, Yonekura Y, Sadato N (2006). Practice makes perfect: the neural substrates of tactile discrimination by Mah-Jong experts include the primary visual cortex. *BMC Neuroscience*.

[56] Merabet LB, Swisher JD, McMains SA (2007). Combined activation and deactivation of visual cortex during tactile sensory processing. *Journal of Neurophysiology*.

[57] Murata A, Gallese V, Kaseda M, Sakata H (1996). Parietal neurons related to memory-guided hand manipulation. *Journal of Neurophysiology*.

[58] Sakata H, Taira M, Murata A, Mine S (1995). Neural mechanisms of visual guidance of hand action in the parietal cortex of the monkey. *Cerebral Cortex*.

[59] Bodegård A, Geyer S, Grefkes C, Zilles K, Roland PE (2001). Hierarchical processing of tactile shape in the human brain. *Neuron*.

[60] Jäncke L, Kleinschmidt A, Mirzazade S, Shah NJ, Freund HJ (2001). The role of the inferior parietal cortex in linking the tactile perception and manual construction of object shapes. *Cerebral Cortex*.

[61] Shikata E, Hamzei F, Glauche V (2001). Surface orientation discrimination activates caudal and anterior intraparietal sulcus in humans: an event-related fMRI study. *Journal of Neurophysiology*.

[62] Fujii T, Tanabe HC, Kochiyama T, Sadato N (2009). An investigation of cross-modal plasticity of effective connectivity in the blind by dynamic causal modeling of functional MRI data. *Neuroscience Research*.

[63] Chabot N, Robert S, Tremblay R, Miceli D, Boire D, Bronchti G (2007). Audition differently activates the visual system in neonatally enucleated mice compared with anophthalmic mutants. *European Journal of Neuroscience*.

[64] Chabot N, Charbonneau V, Laramée ME, Tremblay R, Boire D, Bronchti G (2008). Subcortical auditory input to the primary visual cortex in anophthalmic mice. *Neuroscience Letters*.

[65] Bronchti G, Heil P, Sadka R, Hess A, Scheich H, Wollberg Z (2002). Auditory activation of ’visual’ cortical areas in the blind mole rat (Spalax ehrenbergi). *European Journal of Neuroscience*.

[66] Burton H, Sinclair RJ, McLaren DG (2004). Cortical activity to vibrotactile stimulation: an fMRI study in blind and sighted individuals. *Human Brain Mapping*.

